# Inner Wellbeing: Concept and Validation of a New Approach to Subjective Perceptions of Wellbeing—India

**DOI:** 10.1007/s11205-013-0504-7

**Published:** 2013-11-05

**Authors:** Sarah C. White, Stanley O. Gaines, Shreya Jha

**Affiliations:** 1 Department of Social and Policy Sciences, University of Bath, Claverton Down, Bath, BA2 7AY UK; 2 School of Social Sciences, Brunel University, Uxbridge, UB8 3PH UK

**Keywords:** Subjective wellbeing, International development, Psychosocial, India, Model validation

## Abstract

This paper describes the conceptual development of a multi-domain, psychosocial model of ‘Inner Wellbeing’ (IWB) and assesses the construct validity of the scale designed to measure it. IWB expresses what people think and feel they are able to be and do. Drawing together scholarship in wellbeing and international development it is grounded in field research in marginalised, rural communities in the global South. Results from research in India at two points in time (2011 and 2013) are reported. At Time 1 (n = 287), we were unable to confirm an eight-factor, correlated model as distinct yet interrelated domains. However, at Time 2 (n = 335), we were able to confirm a revised, seven-factor correlated model with economic confidence, agency and participation, social connections, close relationships, physical and mental health, competence and self-worth, and values and meaning (five items per domain) as distinct yet interrelated domains. In particular, at Time 2, a seven-factor, correlated model provided a significantly better fit to the data than did a one-factor model.

## Introduction

The past two decades of publications in this journal and many others bear witness to an explosion of interest in subjective measures of wellbeing and quality of life as ways to assess the impact on individuals of lifestyle choices, behaviour, economics, programmatic interventions and social and political regimes. At their simplest such measures comprise a single ‘global happiness’ or ‘life satisfaction’ question, which asks people to rate how happy they are with their lives as a whole (e.g. Andrews and Withey 1976). The Gallup organisation provides an additional twist in its adaptation of Cantril’s ‘Self-anchoring striving scale,’ as it asks people to rate not only how they are doing at present but also how they predict themselves to be doing in 5 years’ time (Cantril 1965; Gallup 2013). Going up a level of complexity, life satisfaction may be measured through multi-item scales. These may be single construct scales in which all items are designed to reflect aspects of the same construct—global life satisfaction—as in the satisfaction with life scale (Diener et al. 1985). Alternatively multi-domain scales ask people to rate their satisfaction with a range of life domains with the average across the domains being used to produce an overall score, as in the Personal Wellbeing Index (International Wellbeing Group 2006). Measures of life satisfaction are also commonly twinned with a measure of affect balance (weighing up the experience of positive against negative emotions) to produce a subjective wellbeing score (e.g. Diener et al. 1999). It is, however, increasingly evident that measures of affect and of life satisfaction behave in rather different ways, suggesting that they may be better assessed separately from each other (Diener et al. 2010; Graham 2011).

The twin exigencies of seeking maximum comparability with minimum investment mean that subjective wellbeing approaches are deliberately ‘slim’. In practical terms, therefore, with the exception of some means of assessing the affect balance, these measures tend to be quite short and therefore relatively light-weight to administer. Conceptually also, they do not seek to engage with the substance of people’s lives, aiming only to deliver a summary measure of their subjective success.

Critics within the wellbeing literature suggest that subjective wellbeing provides too narrow an understanding of happiness, that beyond the ‘hedonic’ emphasis on pleasure is a ‘eudaemonic’ understanding of the good life in terms of functioning and fulfilment—a life well-lived (Ryan and Deci 2001). The eudaemonic approach leads to fuller conceptualisations of psychological wellbeing. A leading example is Self-Determination Theory, which sees psychological wellbeing as resulting from the ability to meet three basic psychological needs: autonomy, competence, and relatedness (Ryan and Deci 2000). An alternative is the multi-domain approach of Carol Ryff and her colleagues, which conceives of psychological wellbeing in terms of ‘optimal psychological functioning’ (Ryff 1989; Ryff and Keyes 1995).

This paper describes the development of a new concept, ‘Inner Wellbeing’ (IWB), which aims to capture what people think and feel they are able to be and do, and the validation of a scale to assess this. This makes four distinctive contributions to the understanding of subjective dimensions of wellbeing. First, while IWB lies within the eudaemonic tradition, it takes this in a psychosocial direction, which emphasises people’s grounding in a particular material, social, political and cultural context, rather than internal psychological processes. Second, while the dominant approaches were originally developed amongst relatively affluent respondents in the West, the theorisation of IWB deliberately prioritised research undertaken in and on countries of the global south, and the scale was developed through research in marginalised rural communities in Zambia and India. Third, the primary aim of SWB is to produce an abstract summative measure of happiness or satisfaction—or in economic terms, utility—which is as clean as possible of the particularities of the lives from which it is derived. IWB, by contrast, seeks to engage with at least some of the substance of how people are thinking and feeling about different aspects of their lives, so as to understand how their perceptions of different kinds of strength or vulnerability might have a material effect on what they can achieve. Finally, where Subjective Wellbeing ultimately positions people as consumers, rating their happiness or satisfaction with their lives, IWB constructs people in active voice, as it seeks to explore the scope of what people think and feel they are able to be and do.

## Developing the Model of Inner Wellbeing

As an interdisciplinary team combining psychology and international development, our intellectual inspiration is unusually broad. Just as wellbeing engages scholars across the social sciences, so international development is a field of study which spans natural resource management and economics at one end to sociology and social anthropology at the other. While wellbeing appears as a new focus in international development, it also takes forward long established trends to advance recognition of the social and personal in response to the dominance of economics (White 2013). The most obvious of these is the move to recognise poverty and development in multidimensional terms (e.g. Alkire 2002). It also builds on and advances the insights of livelihoods approaches, which emphasise people’s strengths rather than needs and see economic activity as a complex mix of priorities, strategies, influences, activities and alliances which draw on a range of material and social resources (e.g. Carney 1988; Saltmarshe 2002). In economics Amartya Sen (1983a) made a critical shift towards greater recognition of social relations with his argument that famine results not from absolute shortages of food but deprivation of social and economic entitlements. His work on ‘capabilities and functionings’ directly pre-figures the IWB approach, as it relates living standards not simply to what people have, but what they can do and be (Sen 1983b, 2003). Work on participation shows the importance of listening to local perspectives and ensuring that local people participate actively in shaping the change that is to come (e.g. Cornwall 2011). Finally, feminist work on women’s empowerment has brought sensitivity to the impact of personal relationships, self-confidence, how people imagine themselves, and more generally to the politics of the personal (e.g. Rowlands 1997).

While these sources may be seen as the broader ancestry of IWB, its direct line of descent lies in the first author’s participation in the Wellbeing in Developing Countries Research Group (WeD), based at the University of Bath, UK, 2002–2007 (www.welldev.org.uk). WeD was a large, four country (Bangladesh, Ethiopia, Peru and Thailand) comparative study of wellbeing, which comprised both ‘objective’ accounts of livelihoods—characterised as ‘resources and needs’—and a subjective measure of goals and satisfaction, characterised as ‘quality of life’ (Gough and McGregor 2007; Woodcock et al. 2009).^1^ A core outcome of the programme was an understanding of wellbeing as comprising three dimensions—material, relational and subjective (Gough et al. 2007; McGregor 2007; White 2010). While this remains important to our thinking, it is not straightforward to translate into a practical guide for research. Particular items or issues do not slot neatly into one category or another as either material, or relational, or subjective. Instead, almost any issue—such as land or food or marriage—draws together all of material, relational and subjective dimensions (White 2010). Marriage, for example, has material effects in terms of patterns of residence, finance and the production of offspring. Relationally it forms bonds not just between the couple themselves but also their families more widely, as well as being a key indicator of social status. Subjectively marriage has a major impact on how people think and feel about themselves and others. Further, while a wellbeing approach should, in our view, indeed consider material, relational and subjective dimensions, it is not clear that this is peculiar to a focus on wellbeing, as opposed to something common to any sociologically aware research. This signifies a more general difficulty, that WeD’s conception of wellbeing was so broad and encompassing that it was not clear what it excluded, and not easy to see how claims that were made about it might be amenable to empirical analysis, or in Popper’s terms, ‘falsification’ (Popper 1963).

The first step towards IWB, therefore, was the view that research on wellbeing should be ‘risky’—it should be open to being proved wrong. This led to the design of a new research project ‘Wellbeing and Poverty Pathways’ (www.wellbeingpathways.org). This would explore people’s movements into, within and out of poverty using demographic and economic indicators on the one hand and subjective perspectives and reflections on the other. Critically, it would seek to develop a psychosocial model of wellbeing which could be assessed in quantitative terms, and so be amenable to empirical testing as to how much, if anything, it added to more established ‘objective’ measures in explaining poverty dynamics.

The WeD programme had itself produced a subjective quality of life instrument, the WeDQoL (Yamamoto 2008; Woodcock et al. 2009). This defined subjective quality of life as ‘the outcome of the gap between people’s goals and perceived resources… in the context of their culture, values, and experiences of un/happiness (Woodcock et al. 2009: 137). ‘Goals’ were generated through asking people in the study communities what they needed to be happy or to live well.^2^ The data were then subject to psychometric analysis to produce a final list and respondents were asked to rate how necessary they felt each item to be and how satisfied they were with them.

The 44 goals identified for Thailand clearly suggest some culturally specific aspects of wellbeing with items such as ‘having a beautiful house’ ‘spending money wisely,’ ‘[being] satisfied with what you have’, ‘having loving-kindness for others’ and ‘well-behaved children’ (Woodcock et al. 2009: 139–140).^3^ However, the approach as a whole still reproduces the essential subject position noted above in relation to Subjective Wellbeing, of respondents as consumers who have objects of desire and are satisfied or dissatisfied with their ability to secure them. This is emphasised by the character of the ‘goals’ themselves, many of which are material goods. On the other hand, where the items concern personal qualities or relationships, such as those cited above, the object-relationship seems a rather uncomfortable fit. We were also uneasy with the heterogeneity of the list (could such different kinds of things be meaningfully treated as equivalent?) and its simple empiricism, outside of any theoretical context. We therefore decided that we needed to look further.

Our search for an approach to wellbeing that had been developed in the context of the global South led us to the Sri Lanka-based research programme, the Psycho-social Assessment of Development and Humanitarian Intervention (PADHI) and their ‘social justice approach to wellbeing’ (PADHI 2009). Like the WeDQoL, PADHI’s approach to wellbeing was based on qualitative work asking people what it meant to live well. Their data analysis, however, led PADHI to identify five domains of wellbeing which they describe in active terms. Thus for PADHI people ‘experience wellbeing when they are able to: access valued physical, material, and intellectual resources; experience competence and self-worth; exercise participation; build social connections; and enhance physical and psychological wellness’ (PADHI 2009: 13). While these domains arose from the data, they also clearly carry the marks of the development studies scholarship outlined above. We therefore largely adopted this identification of domains of wellbeing, but made two amendments. First, we added two domains. Research in Bangladesh, partly under the WeD programme but also preceding this, had shown that social and political connections are critical for enabling poor people to gain access to resources (Devine 2002, 2007). Wellbeing scholarship more generally, reinforced by exploratory work on quality of life under WeD, also points, however, to the importance of close family relationships to personal wellbeing. We felt that this more intimate aspect of close family relationships was distinct from more political social connections, and so required a different domain. In addition, research on religion and wellbeing in India and Bangladesh had showed how closely intertwined these were (Devine and White 2013; White et al. 2012). We therefore added a domain on ‘values and meaning’ to reflect the importance of religion and the spiritual. The second amendment is that we split the ‘access to resources’ domain in two. One became a subjective assessment of the local environment and the other focused directly on economic confidence. This followed extensive qualitative field testing which showed how much importance people gave to their economic circumstances in describing their experience of wellbeing. As described below, the local environment domain was not supported in the first round of field research and was ultimately dropped from the model. IWB therefore retains seven domains: economic confidence; agency and participation; social connections; close relationships; physical and mental health; competence and self-worth; values and meaning.

## Methodology

Ethics approval was given before commencement of the study by the authors’ academic institutions. Field research was undertaken in marginalised rural communities in Zambia (Chiawa) and India (Sarguja district, Chhattisgarh state). Two rounds of fieldwork were undertaken in each place, in Zambia August–November 2010 and August–October 2012; in India February–May 2011 and February–June 2013. The 2 year gap between field trips was designed to provide a mini panel series. The seasonality of rural livelihoods made it important to hold each round of data collection in each place at the same time of year. The main research instrument was a survey which comprised three sections: an opening section on demographics and health; the central IWB section; and a final section on livelihoods and access to state services. The need to reflect local realities, coupled with learning and reflection between field research periods, meant that there was some change in specific survey items over time. We report only on the IWB section in this paper. The survey was conducted with husbands and wives (interviewed separately) and single women heading households. We included single women heading households as they are commonly found to experience particular vulnerabilities. In almost all cases they had previously been married, and were either widowed or divorced. Considerations of space mean that we report only on the India research here. Analysis of Zambia findings will be presented in a different paper.

Our Indian research location was four villages in the ‘remote’ hill and forest areas of northern Chhattisgarh (Central India). We were supported in the field by a local non-governmental organisation, Chaupal, who also helped us recruit four local research assistants (two men and two women). The field research was led by the Wellbeing Pathways research officer (the third author). Most of the people in our research villages were Adivasi, ethnic minority communities classified as ‘Scheduled Tribes’ under British-colonial administration, which have a long history of problematic relationships with outsiders (Sundar 2007).^4^ These are extremely poor communities, amongst whom hunger was commonplace until recently.^5^ Literacy levels are very low, with more than half of our respondents reporting no schooling at all and a further 20 % being able only to write their own names. The mainstay of the economy is rain-fed agriculture, with most people doing some farming, supplemented by casual labour and gathering of non-timber forest products. Participation was voluntary, no financial incentives were offered. However, there is no doubt that the goodwill that Chaupal enjoyed in our research villages was crucial to people’s readiness to talk to us.

There is not space here to discuss in detail the fieldwork methodology and the challenges this context posed to many of the academic conventions that govern the collection of data for psychometric analysis. These are described more fully elsewhere (White and Jha, forthcoming). It is nonetheless important to appreciate some of this as a background to the next section, which describes how—particularly at Time 1—the results did not conform to normally expected patterns.

IWB items were tested and re-tested through an extensive period of qualitative grounding and piloting, to ensure their relevance and intelligibility within the particular context. Once finalised, questionnaires were translated into Hindi and completed through interview, with informants giving oral responses which were noted down by members of the research team. The data was then inputted in English into excel, and subsequently cleaned and transposed for analysis into SPSS. Informed consent was provided orally, as was debriefing on conclusion of the survey. Perhaps the most significant challenge was in dealing with the abstract and generalised form usually taken by subjective survey questions. Our respondents had difficulty making sense of these, requiring instead questions in a form that was much more specific and tangible. Similarly, a range of graduated answers had to be offered in verbal form, rather than a numerical response scale. This relates in part to the structure of the language itself: English is relatively direct and abstract; Hindi is much more indirect and concrete. It also reflects the kind of exposure people have had. Unfortunately we have not been able to find much discussion of this in the literature, perhaps in part because psychological surveys still tend to be undertaken with rather highly schooled populations. There is, however, some indication of similar challenges in Biswas-Diener and Diener’s (2001) account of their study of subjective wellbeing amongst poor people in Calcutta.^6^

### Time 1

The goal of our study at Time 1 was to test our initial hypothesis that an eight factor model of IWB (comprising economic confidence, local environment, agency and participation, social connections, close relationships, physical and mental health, competence and self-worth, values and meaning) will provide a significantly better fit to data on item score correlations than will a one-factor model of IWB. However, as we discuss below, we also found it necessary to test the revised hypothesis that an eight-factor model of IWB as *un*correlated domains will provide significantly better fit to data on item score correlations than will a one-factor model of IWB. We tested the hypotheses via LISREL 9.1 (Joreskog and Sorbom 2012a).

A total of 158 married men, 156 married women and 26 women heading households participated in the Time 1 study. The mean age of respondents was 41 years (SD = 12 years). The scale contained 32 items (4 items per domain), designed to measure the eight IWB domains. Each item was scored along a 5-point, Likert-type scale, with the content of the scale points varying from item to item. Reverse-worded items were rescored so that for all items, higher scores reflected higher levels of the IWB domain in question. A complete set of items for Time 1 is presented in Table 1.

**Table 1:** Set of items measuring dimensions of inner wellbeing at Time 1

*Economic*
1. How well would you say you are able to live (*managing*) at present?
2. If guests come do you feel you can look after them in the proper way?
3. Do you feel that people around you have got ahead of you?
4. Do you feel that your children will have a better life than you have had?
*Local environment*
1. What percentage of people in this village are helpful (to you)?
2. Do you feel you face (gender, caste, economic) discrimination in this community?
3. Do you feel this village has the services that will help you progress?
4. If the forest resources decline how big an impact will it have on your life?
*Agency and participation*
1. If there is a village meeting do you have an opportunity to voice your opinion?
2. If official decisions are made that affect you badly, do you feel that you have power to change them?
3. Do you feel that you are heard? (NB outside the family)
4. How confident do you feel that (along with others) you will be able to bring change to your community?
Social connections
1. Do you know the kind of people who can help you get things done?
2. When there is news in the community, how many people get to know about before you do?
3. Do you feel that there are some people who would like to cause you harm?
4. Do you feel there are people beyond your immediate family who you’ll be able to count on even through bad times?
*Close/family relationships*
1. Do you feel that there is unity in your family?
2. If problems arise in your family, how often are you able to discuss and sort them out?
3. How often is it that your family requires you to do things that you don’t want?
4. How much do people in your house care for you?
*Physical and mental health*
1. Do you ever have trouble sleeping?
2. Compared to other (men/women) of your age, how fit and healthy would you say you are?
3. Do you suffer from tension?
4. Do you have enough time to rest and relax?
*Competence and self*-*worth*
1. To what extent have you been able to overcome life’s difficulties?
2. If something goes wrong to what extent do you hold yourself responsible (take the blame)?
3. How much have you accomplished of what you wanted to accomplish at this point in your life?
4. Looking to the future, how confident do you feel that you will be able to fulfil your responsibilities?
*Values and meaning*
1. How satisfied are you with the place you are able to make for religion in your life?
2. How far would you say you feel peace in your heart at the end of the day?
3. To what extent would you say that you live in fear of harm from witchcraft, evil gaze, magic?
4. To what extent do you feel that life has been good to you?

## Results and Discussion

### Normalisation of Item Scores

Data concerning one or more IWB items were missing for 53 individuals, leaving a final total of 287 individuals for Time 1 analyses. We began our analyses by entering the raw data into PRELIS 9.1.^7^ Means, standard deviations, and skewness and kurtosis statistics for item scores at Time 1 are shown in Table 2. For two items, skewness was >2.30 in absolute value; and for seven items, kurtosis was >2.30 in absolute value.^8^

**Table 2:** Means, standard deviations, skewness, and kurtosis statistics before normalisation of item scores, Time 1 (n = 287)

Item	Mean	SD	Skewness	Kurtosis
EC1	3.165	0.872	−0.003	0.523
EC2	3.519	1.044	−0.145	−0.924
EC3	2.410	0.825	0.513	0.763
EC4	3.294	1.277	−0.142	−1.018
EV1	2.900	1.366	0.398	−1.141
EV2	4.374	1.080	−1.930	3.003
EV3	2.682	1.115	0.344	−0.525
EV4	1.324	0.866	2.828	7.191
AP1	2.603	1.505	0.509	−1.138
AP2	2.490	1.424	0.643	−0.898
AP3	2.597	1.420	0.557	−1.003
AP4	2.728	1.264	0.435	−0.744
SC1	2.129	1.225	0.808	−0.495
SC2	2.540	1.091	0.077	−0.694
SC3	4.346	0.903	−1.641	2.711
SC4	2.159	1.189	1.161	0.626
CR1	4.589	0.805	−2.189	4.647
CR2	4.604	0.810	−2.171	4.203
CR3	4.155	0.976	−1.561	2.634
CR4	4.598	0.797	−2.105	4.010
H1	3.974	1.111	−1.387	1.462
H2	2.524	0.860	0.416	0.369
H3	2.838	1.521	0.008	−1.496
H4	2.971	1.278	0.439	−1.056
SW1	2.784	1.077	0.296	−0.347
SW2	2.671	1.312	−0.018	−1.296
SW3	2.563	1.043	0.445	−0.133
SW4	3.480	1.281	−0.306	−0.962
VM1	4.729	0.689	−2.848	8.633
VM2	3.312	1.237	−0.050	−0.999
VM3	2.800	1.676	0.141	−1.651
VM4	3.829	1.034	−0.589	−0.082

*EC* economic confidence, *EV* local environment, *AP* agency and participation, *SC* social connections, *CR* close relationships, *H* physical and mental health, *SW* competence and self-worth, *VM* values and meaning

On entering the raw data into PRELIS 9.1, we normalised all item scores. As Table 3 indicates, this all but eliminated instances of severe kurtosis. Subsequently, using PRELIS 9.1, we calculated a correlation matrix based on the normalised scores at Time 1^9^ for entry into the main LISREL 9.1 routine.

**Table 3:** Means, standard deviations, skewness, and kurtosis statistics after normalisation of item scores, Time 1 (n = 287)

Item	Mean	SD	Skewness	Kurtosis
EC1	3.160	0.862	0.013	0.193
EC2	3.523	1.040	−0.124	−0.617
EC3	2.415	0.801	0.102	0.008
EC4	3.300	1.257	−0.163	−0.793
EV1	2.868	1.342	−0.036	−0.832
EV2	4.366	1.104	−1.097	−0.175
EV3	2.763	1.100	0.066	−0.467
EV4	1.265	0.780	2.482	4.793
AP1	2.596	1.500	0.163	−1.289
AP2	2.498	1.431	0.204	−1.125
AP3	2.641	1.412	0.102	−1.062
AP4	2.798	1.280	0.025	−0.817
SC1	2.174	1.239	0.463	−0.823
SC2	2.589	1.077	0.145	−0.501
SC3	4.352	0.892	−0.777	−0.417
SC4	2.111	1.126	0.319	−0.707
CR1	4.613	0.776	−1.525	1.066
CR2	4.631	0.773	−1.620	1.352
CR3	4.136	0.992	−0.456	−0.607
CR4	4.634	0.745	−1.579	1.228
H1	3.972	1.093	−0.350	−0.645
H2	2.568	0.858	0.085	−0.041
H3	2.829	1.520	0.211	−1.183
H4	2.941	1.271	−0.033	−0.676
SW1	2.784	1.049	0.049	−0.367
SW2	2.592	1.295	0.272	−0.836
SW3	2.530	1.034	0.122	−0.413
SW4	3.512	1.273	−0.280	−0.864
VM1	4.739	0.662	−2.096	3.041
VM2	3.321	1.218	−0.177	−0.723
VM3	2.868	1.659	0.169	−1.486
VM4	3.812	1.037	−0.301	−0.680

*EC* economic confidence, *EV* local environment, *AP* agency and participation, *SC* social connections, *CR* close relationships, *H* physical and mental health, *SW* competence and self-worth, *VM* values and meaning

### Confirmatory Factor Analyses of Inner Wellbeing Questionnaire

We evaluated the goodness-of-fit of a one-factor model, via the maximum likelihood and invoking the ridge option (using LISREL 9.1; Joreskog and Sorbom 2012a).^10^ Results of the confirmatory factor analysis indicated that a one-factor model provided adequate fit to the correlational data (χ^2^ = 262.24, *df* = 495, *NS*; χ^2^/*df* ratio = 0.53; standardised root mean square residual = 0.04; adjusted goodness-of-fit index = 0.93). Moreover, as indicated in Table 4, all loadings were positive; and we obtained significant or marginal loadings for most of the items (i.e., 24 of the 32 loadings were significant, with *p*’s < .05 or lower; an additional 3 loadings were marginal, with *p*’s < .10).

**Table 4:** Loadings for all items on one hypothesised factor, Time 1 (n = 287)

Item	Loading
EC1	0.530**
EC2	0.524**
EC3	0.401**
EC4	0.437**
EV1	0.289**
EV2	0.119
EV3	0.275*
EV4	0.138
AP1	0.365**
AP2	0.413**
AP3	0.484**
AP4	0.479**
SC1	0.390**
SC2	0.366**
SC3	0.052
SC4	0.287**
CR1	0.199^+^
CR2	0.122
CR3	0.145
CR4	0.155
H1	0.221*
H2	0.383**
H3	0.280*
H4	0.319**
SW1	0.518**
SW2	0.023
SW3	0.480**
SW4	0.476**
VM1	0.185^+^
VM2	0.395**
VM3	0.339**
VM4	0.525**

*EC* economic confidence, *EV* local environment, *AP* agency and participation, *SC* social connections, *CR* close relationships, *H* physical and mental health, *SW* competence and self-worth, *VM* values and meaning

^+^ *p* < .10; * *p* < .05; ** *p* < .01

Subsequently, we attempted to evaluate the goodness-of-fit concerning an eight-factor model with correlated factors.^11^ Unfortunately, the confirmatory factor analysis of eight-factor, intercorrelated model yielded an error message indicating that one or more parameters could not be identified.^12^

Finally, we evaluated the goodness-of-fit of a revised version of the eight-factor model in which the factor intercorrelations were fixed at 0.00, rather than freed. Results of the confirmatory factor analysis indicated that an eight-factor, non-intercorrelated model provided adequate fit to the correlational data (χ^2^ = 384.64, *df* = 488, *NS*; χ^2^/*df* ratio = 0.79; standardised root mean square residual = 0.07; adjusted goodness-of-fit index = 0.90). For seven of the factors, most (if not all) of the loadings (shown in Table 5) were positive and were significant or marginal. However, for the local environment factor, *none* of the loadings turned out to be significant.^13^

**Table 5:** Loadings of items on eight non-intercorrelated factors, Time 1 (n = 287)

Item	Factor							
	EC	EV	AP	SC	CR	H	SW	VM
EC1	0.602**							
EC2	0.597**							
EC3	0.467**							
EC4	0.447**							
EV1		0.292						
EV2		0.232						
EV3		−0.220						
EV4		0.070						
AP1			0.522**					
AP2			0.577**					
AP3			0.595**					
AP4			0.559**					
SC1				0.516**				
SC2				0.452**				
SC3				−0.004				
SC4				0.456**				
CR1					0.600**			
CR2					0.559**			
CR3					0.146			
CR4					0.467**			
H1						0.477**		
H2						0.338*		
H3						0.470**		
H4						0.280^+^		
SW1							0.593**	
SW2							0.198	
SW3							0.583**	
SW4							0.349*	
VM1								0.408**
VM2								0.520**
VM3								0.348*
VM4								0.553**

*EC* economic confidence, *EV* local environment, *AP* agency and participation, *SC* social connections, *CR* close relationships, *H* physical and mental health, *SW* competence and self-worth, *VM* values and meaning

^+^ *p* < .10; * *p* < .05; ** *p* < .01

A direct comparison of the one-factor and eight-factor, non-intercorrelated models revealed that, although both models were plausible (i.e., neither model could be rejected), the one-factor model was associated with significantly *lower* error than was the eight-factor, non-intercorrelated model (difference in χ^2^ = −121.30, difference in *df* = 7, *p* < .01). This is the opposite to what one would expect, as usually the model with the higher number of freed parameters to be estimated and, hence, lower number of *df* (i.e., the eight-factor, non-intercorrelated model) would provide a better fit than would the model with the lower number of freed parameters to be estimated and, hence, higher number of *df* (i.e., the one-factor model; see Brown 2006). All in all, our hypothesis (whether initial or revised) that an eight factor model of IWB would provide a better fit than a one factor model was not supported.

### Discussion

The immediate response to these findings might be to conclude that a unidimensional model of IWB should be preferred over a multidimensional one. Setting that on one side for a moment, we reflected that while seven of the domains were supported by confirmatory factor analysis, the local environment domain was not. At first we were puzzled by this: people in our research villages are economically dependent on forest products, and the existence or otherwise of local infrastructure and services is clearly important in their daily lives. On further reflection, however, we realised that there were a number of conceptual difficulties with the environment domain. The first was that the environment was by definition common to all—a strong effect on everyone would bring little variability in response. The second was that the items in that domain referred to quite different external factors (helpfulness of the community; caste or gender discrimination; amenities and services; forest decline) so there was no reason that they *should* come together as a single factor. The third and most fundamental problem with the domain was that it was external in its orientation, rather than asking people to reflect on what they themselves felt able to do or be. Since statistical results and conceptual reflection led in the same direction, we decided to jettison the local environment domain. Furthermore, drawing on our field experience at Time 1 we also decided to re-work some items in the other domains, and add an additional item per domain to help boost reliability scores. With a revised survey containing only seven domains of wellbeing we therefore hypothesised that we might not only obtain support for seven correlated factors but we might also obtain comparatively better fit for a seven-factor, intercorrelated model than we obtain for a one-factor model.

### Time 2

A total of 371 individuals (177 married men, 171 married women, and 23 single women heading households) participated in the Time 2 study. Of these 187 had also taken part at Time 1, 189 were newly recruited in Time 2. Although this sample attrition was disappointing to us, the reasons for it were largely positive: people were busier because of the greater availability of economic opportunities than at Time 1. The average age of participants at Time 2 was 42 years (SD = 12.5 years).

Participants at Time 2 completed a set of 35 items (5 items per domain), designed to measure the seven IWB domains. A total of 21 items from Time 1 were retained, though some with minor rewording. These included all four items from the Time 1 economic confidence and agency and participation scales; three items from the Time 1 social connections,^14^ competence and self-worth, and values and meaning scales; and two items from the close relationships and physical and mental health scales. 14 new items were added for Time 2, to make up 5 items in each domain. As before, each item was scored along a 5-point, Likert-type scale, with the content of the scale points varying from item to item. Reverse-worded items were managed through the scoring of answers so there was no need for recoding. A complete set of items for Time 2 is presented in Table 6.

**Table 6:** Set of items measuring dimensions of inner wellbeing at Time 2

*Economic confidence*
1. How well would you say you are managing economically at present?
2. If guests come do you feel you can look after them in the proper way?
3. Do you feel that people around are richer than you?
4. How confident do you feel that your children will have a better life than you have had?
5. How well could you manage if something bad were to happen (e.g. illness in the family)?
*Agency and participation*
1. If there is a village meeting do you have an opportunity to voice your opinion?
2. If official decisions are made that affect you badly, do you feel that you have power to change them?
3. Do feel that you are heard? (beyond family)
4. How confident do you feel that the community can get together to take action?
5. How much freedom do you have to make your own decisions about the things that matter to you?
*Social connections*
1. Do you know the kind of people who can help you get things done?
2. When do you get to hear about gossip in the community?
3. How much can you trust people beyond your immediate family to be with you through bad times?
4. What proportion of people in the community are helpful (to you)?
5. Even when others are around, how often do you feel alone?
6. Do you feel that there are some people who would like to cause you harm?^a^
*Close relationships*
1. How well do you get along amongst yourselves?
2. If there is a problem in your family how easily can you sort it out?
3. When your mind/heart is troubled/heavy, do you feel there is someone that you can go to?
4. How much do people in your house care for you?
5. How uneasy are you made by the amount of violence in your home?
*Physical and mental health*
1. Do you ever have trouble sleeping?
2. Do you have the strength that you need for your daily work?
3. Do you suffer from tension?
4. How much do you worry about your health?
5. Do you feel that you have to do more work than you are able to?
*Competence and self*-*worth*
1. How well have you been able to overcome life’s difficulties?
2. How far do you feel you are able to help other people?
3. To what extent do you have faith in yourself?
4. How close would you say you are to accomplishing what you hoped for at this time of your life?
5. Looking to the future, how confident do you feel that you will be able to fulfil your responsibilities?
*Values and meaning*
1. How well are your gods and goddesses looking after you?
2. How lucky have you been in your life?
3. How much peace do you experience peace in your mind/heart?
4. To what extent would you say that you fear harm from witchcraft or evil powers?
5. To what extent do you feel that life has been good to you?

^a^This item measures negative social connections and so was not included in any of the analyses reported in this paper

Data concerning one or more IWB items were missing for 31 individuals, leaving a final total of 335 individuals for Time 2 analyses. As was the case at Time 1, we began our Time 2 analyses by entering the raw data into PRELIS 9.1 (Joreskog and Sorbom 2012b). Means, standard deviations, and skewness and kurtosis statistics for item scores at Time 2 are shown in Table 7. For two items, skewness was >2.30 in absolute value; and for two items, kurtosis was >2.30 in absolute value.

**Table 7:** Means, standard deviations, skewness, and kurtosis statistics before normalisation of item scores, Time 2 (n = 335)

Item	Mean	SD	Skewness	Kurtosis
EC1	3.070	0.981	0.066	−0.520
EC2	3.418	0.981	−0.157	−0.655
EC3	2.392	1.136	0.113	−1.195
EC4	3.259	1.394	−0.139	−1.234
EC5	2.402	0.911	0.003	−0.160
AP1	2.844	1.586	0.255	−1.476
AP2	2.525	1.448	0.520	−1.068
AP3	3.111	1.376	−0.037	−1.095
AP4	2.384	1.398	0.650	−0.850
AP5	3.989	1.219	−1.248	0.583
SC1	1.906	1.371	1.214	0.019
SC2	2.881	1.326	0.066	−0.900
SC3	3.127	1.451	−0.020	−1.338
SC4	3.472	1.350	−0.261	−1.192
SC5	4.024	1.284	−1.280	0.505
CR1	4.746	0.683	−3.171	10.687
CR2	4.089	0.915	−1.197	1.617
CR3	2.832	1.649	0.139	−1.636
CR4	4.741	0.737	−3.209	10.483
CR5	2.750	1.812	0.200	−1.808
H1	3.751	1.386	−0.946	−0.391
H2	3.189	1.052	−0.230	−0.411
H3	2.507	1.458	0.210	−1.537
H4	2.678	0.910	0.508	−0.148
H5	4.263	1.167	−1.616	1.632
SW1	3.024	1.033	0.174	−0.142
SW2	3.265	1.298	−0.300	−1.067
SW3	3.757	1.230	−0.521	−0.830
SW4	2.716	1.016	0.249	0.128
SW5	3.771	1.183	0.524	−0.738
VM1	3.981	1.259	−1.019	−0.112
VM2	3.485	1.086	−0.280	−0.589
VM3	3.295	1.291	−0.190	−1.016
VM4	2.981	1.637	−0.096	−1.638
VM5	3.491	0.947	−0.291	−0.011

*EC* economic confidence, *EV* local environment, *AP* agency and participation, *SC* social connections, *CR* close relationships, *H* physical and mental health, *SW* competence and self-worth, *VM* values and meaning

Upon entering the raw data into PRELIS 9.1, we normalised all item scores. As Table 8 indicates the normalisation procedure at Time 2 resulted in a reduction from two items to one item with severe skewness (the number of items with severe kurtosis was unchanged at two). Subsequently, using PRELIS 9.1, we calculated a correlation matrix based on the normalised scores at Time 2^15^ for entry into the main LISREL 9.1 routine.

**Table 8:** Means, standard deviations, skewness, and kurtosis statistics after normalisation of item scores, Time 2 (n = 335)

Item	Mean	SD	Skewness	Kurtosis
EC1	3.063	0.966	0.010	−0.259
EC2	3.433	0.985	−0.086	−0.419
EC3	2.382	1.128	0.270	−0.770
EC4	3.272	1.412	−0.204	−1.054
EC5	2.415	0.921	0.025	−0.339
AP1	2.866	1.604	−0.001	−1.414
AP2	2.549	1.469	0.240	−1.207
AP3	3.110	1.381	−0.095	−1.018
AP4	2.406	1.394	0.323	−1.091
AP5	3.970	1.221	−0.479	−0.841
SC1	1.925	1.372	0.945	−0.597
SC2	2.904	1.352	0.058	−0.954
SC3	3.122	1.437	−0.130	−1.098
SC4	3.478	1.351	−0.332	−0.937
SC5	4.009	1.284	−0.606	−0.912
CR1	4.758	0.660	−2.229	3.673
CR2	4.078	0.925	−0.408	−0.474
CR3	2.800	1.648	0.165	−1.451
CR4	4.761	0.703	−2.483	4.805
CR5	2.749	1.802	0.307	−1.647
H1	3.737	1.398	−0.346	−1.112
H2	3.215	1.050	−0.055	−0.376
H3	2.513	1.460	0.446	−0.966
H4	2.696	0.904	0.151	−0.095
H5	4.218	1.185	−0.895	−0.545
SW1	3.063	1.026	−0.038	−0.245
SW2	3.281	1.287	−0.110	−0.792
SW3	3.779	1.230	−0.478	−0.804
SW4	2.719	1.032	0.017	−0.296
SW5	3.791	1.173	−0.429	−0.770
VM1	4.021	1.242	−0.686	−0.763
VM2	3.510	1.094	−0.176	−0.579
VM3	3.343	1.292	−0.173	−0.872
VM4	2.991	1.644	0.118	−1.431
VM5	3.504	0.941	−0.099	−0.302

*EC* economic confidence, *EV* local environment, *AP* agency and participation, *SC* social connections, *CR* close relationships, *H* physical and mental health, *SW* competence and self-worth, *VM* values and meaning

### Confirmatory Factor Analyses of Inner Wellbeing Questionnaire

We evaluated the goodness-of-fit of a one-factor model, via the maximum likelihood and invoking the ridge option (using LISREL 9.1; Joreskog and Sorbom 2012a).^16^ Results of the confirmatory factor analysis indicated that a one-factor model provided adequate fit to the correlational data (χ^2^ = 384.43, *df* = 594, *NS*; χ^2^/*df* ratio = 0.65; standardised root mean square residual = 0.05; adjusted goodness-of-fit index = 0.92). Moreover, as indicated in Table 9, all loadings were positive; and we obtained significant loadings for all 35 items (with *p*’s < .05 or lower).

**Table 9:** Loadings for all items on one hypothesised factor, Time 2 (n = 335)

Item	Loading
EC1	0.522**
EC2	0.535**
EC3	0.423**
EC4	0.433**
EC5	0.493**
AP1	0.427**
AP2	0.350**
AP3	0.446**
AP4	0.385**
AP5	0.349**
SC1	0.374**
SC2	0.380**
SC3	0.448**
SC4	0.449**
SC5	0.455**
CR1	0.245**
CR2	0.205*
CR3	0.226**
CR4	0.287**
CR5	0.182*
H1	0.209*
H2	0.337**
H3	0.324**
H4	0.337**
H5	0.282**
SW1	0.504**
SW2	0.429**
SW3	0.566**
SW4	0.425**
SW5	0.457**
VM1	0.298**
VM2	0.500**
VM3	0.508**
VM4	0.348**
VM5	0.521**

*EC* economic confidence, *EV* local environment, *AP* agency and participation, *SC* social connections, *CR* close relationships, *H* physical and mental health, *SW* competence and self-worth, *VM* values and meaning

* *p* < .05; ** *p* < .01

Afterwards, we evaluated the goodness-of-fit concerning a seven-factor model with correlated factors.^17^ Results of the confirmatory factor analysis indicated that a seven-factor, correlated model provided adequate fit to the correlational data (χ^2^ = 288.95, *df* = 567, *NS*; χ^2^/*df* ratio = 0.51; standardised root mean square residual = 0.04; adjusted goodness-of-fit index = 0.94). Moreover, as indicated in Table 10, all loadings were positive; and we obtained significant loadings for all 35 items on their respective factors (with *p*’s < .05 or lower). Finally (and consistent with our hypotheses), the seven-factor intercorrelated model provided a significantly better fit to the correlational data than did the one-factor model (difference in χ^2^ = 95.48, difference in *df* = 27, *p* < .01).

**Table 10:** Loadings of items on seven intercorrelated factors, Time 2 (n = 335)

Item	Factor						
	EC	AP	SC	CR	H	SW	VM
EC1	0.632**						
EC2	0.644**						
EC3	0.493**						
EC4	0.483**						
EC5	0.556**						
AP1		0.572**					
AP2		0.440**					
AP3		0.612**					
AP4		0.502**					
AP5		0.293**					
SC1			0.450**				
SC2			0.468**				
SC3			0.577**				
SC4			0.585**				
SC5			0.459**				
CR1				0.434**			
CR2				0.420**			
CR3				0.260*			
CR4				0.497**			
CR5				0.283*			
H1					0.470**		
H2					0.528**		
H3					0.564**		
H4					0.447**		
H5					0.425**		
SW1						0.545**	
SW2						0.451**	
SW3						0.604**	
SW4						0.469**	
SW5						0.503**	
VM1							0.394
VM2							0.561
VM3							0.598
VM4							0.376
VM5							0.617

*EC* economic confidence, *EV* local environment, *AP* agency and participation, *SC* social connections, *CR* close relationships, *H* physical and mental health, *SW* competence and self-worth, *VM* values and meaning

* *p* < .05; ** *p* < .01

Inspection of the matrix of interfactor correlations^18^ shown in Table 11, revealed that all interfactor correlations were positive; and 17 of the 21 correlations were significant, with *p*’s < .05 or lower (an additional 3 correlations were marginal, with *p*’s < .10). One factor score correlation (i.e., between agency/participation and social connections) was so high that it approached 1.00; such a high absolute value, if considered out of context, would raise the question of whether these two dimensions should be interpreted as part of a single factor (see Nunnally and Bernstein 1994). However, the standardised factor score correlations that are generated via confirmatory factor analysis tend to be substantially higher in magnitude than one would expect from scale correlations that are based on simple sums of item scores.^19^ Indeed, zero-order correlations among summed scale scores^20^ were substantially lower than were standardized factor score correlations among all factors (including the correlation between agency/participation and social connections).

**Table 11:** Zero-order correlations among standardised factor scores on seven inner wellbeing dimensions, Time 2 (n = 335)

Item	Correlations						
	EC	AP	SC	CR	H	SW	VM
EC	1.000						
AP	0.597**	1.000					
SC	0.540**	0.949**	1.000				
CR	0.339*	0.347*	0.605**	1.000			
H	0.460**	0.178	0.389**	0.328**	1.000		
SW	0.813**	0.679**	0.711**	0.569**	0.507**	1.000	
VM	0.811**	0.486**	0.487**	0.422**	0.644**	0.815**	1.000

*EC* economic confidence, *EV* local environment, *AP* agency and participation, *SC* social connections, *CR* close relationships, *H* physical and mental health, *SW* competence and self-worth, *VM* values and meaning

* *p* < .05; ** *p* < .01

Finally, we evaluated the goodness-of-fit of a seven-factor model in which the factor intercorrelations were fixed at 0.00, rather than freed. Results of the confirmatory factor analysis indicated that a seven-factor, non-intercorrelated model generally provided adequate fit to the correlational data, although the adjusted goodness-of-fit index was slightly below the desired level of 0.90 (χ^2^ = 544.33, *df* = 588, *NS*; χ^2^/*df* ratio = 0.93; standardised root mean square residual = 0.08; adjusted goodness-of-fit index = 0.88).^21^ Moreover, as indicated in Table 12, all loadings were positive; and we obtained significant loadings for 34 of the 35 items on their respective factors (with *p*’s < .05 or lower). However (and consistent with hypotheses), the seven-factor uncorrelated model provided a significantly *worse* fit than did the one-factor model (difference in χ^2^ = −173.02, difference in *df* = 6, *p* < .01). Although we did not hypothesise a significant difference between the seven-factor uncorrelated versus seven-factor correlated models, we also found that the seven-factor correlated model provided significantly better fit to the correlational data than did the seven-factor uncorrelated model (difference in χ^2^ = 283.38, difference in *df* = 21, *p* < .01).

**Table 12:** Loadings of items on seven uncorrelated factors, Time 2 (n = 316)

Item	Factor						
	EC	AP	SC	CR	H	SW	VM
EC1	0.632**						
EC2	0.653**						
EC3	0.520**						
EC4	0.487**						
EC5	0.548**						
AP1		0.500**					
AP2		0.536**					
AP3		0.592**					
AP4		0.561**					
AP5		0.168					
SC1			0.385**				
SC2			0.473**				
SC3			0.626**				
SC4			0.603**				
SC5			0.359**				
CR1				0.452**			
CR2				0.475**			
CR3				0.042			
CR4				0.531**			
CR5				0.317**			
H1					0.513**		
H2					0.500**		
H3					0.565**		
H4					0.376**		
H5					0.387**		
SW1						0.535**	
SW2						0.424**	
SW3						0.573**	
SW4						0.488**	
SW5						0.521**	
VM1							0.453**
VM2							0.559**
VM3							0.614**
VM4							0.381**
VM5							0.531**

*EC* economic confidence, *EV* local environment, *AP* agency and participation, *SC* social connections, *CR* close relationships, *H* physical and mental health, *SW* competence and self-worth, *VM* values and meaning

* *p* < .05; ** *p* < .01

### Internal Consistency of Seven Inner Wellbeing Scales

Until this point our analyses have emphasised the importance of construct validity as the means to test our model of IWB. However, a related psychometric property that complements construct validity is *internal consistency*, or the extent to which knowledge of scores on a given item within a scale informs one regarding scores on other items within that scale (Carmines and Zeller 1979). The most popular indicator of internal consistency is Cronbach’s α (Brown 2006).

Based on the number of items and the matrix of zero-order correlations among the items within each of the seven IWB scales at Time 2, we found that standardised Cronbach’s αs were 0.70 for economic, 0.59 for agency/participation, 0.63 for social connections, 0.43 for close relationships, 0.61 for physical/mental health, 0.64 for competence/self-worth, and 0.64 for values/meaning (average Cronbach’s α = 0.61). Given the relatively small number of items per scale, we concluded that with the exception of the close relationships scale, the IWB scales were internally consistent.^22^

### Discussion

The Time 2 results indicate that we have successfully developed an IWB scale that clearly measured seven distinct, yet interrelated, domains amongst our research participants in rural India. Not only did our seven-factor correlated model provide adequate fit to the correlational data, but the seven-factor correlated model also provided significantly better fit to the correlational data than did a one-factor model. Furthermore, within the seven-factor intercorrelated model, every item at Time 2 loaded significantly on its hypothesized factor. Thus, at Time 2, we obtained considerable empirical support for the conceptual model of IWB.

Why were we unable to reject the one-factor model of IWB, either at Time 1 or at Time 2? Although it is tempting to view confirmatory factor analysis as a statistical contest involving two or more models actual results of confirmatory factor analyses rarely yield such clear zero sum results. Rather, results of confirmatory factor analyses are typically nuanced, involving the *relative* fit of one model versus one or more competing models.^23^ We interpret the present results as suggesting that IWB is a coherent construct on which individuals differ (Time 1 and Time 2 results); yet IWB is a complex construct, distinguished by multiple dimensions on which scores covary (Time 2).

Finally, why was the internal consistency so low for the close relationships scale? The answer to this lies outside the realm of statistics. First, close relationships is a part of life that people in our research communities were not used to discussing in direct terms, especially when questioned by strangers. We therefore struggled to devise appropriate items. Second, this domain seemed to be governed by particularly powerful norms which resulted in a strong social desirability bias—whatever people’s actual experience, family unity was what should be projected.^24^ This was reflected in very high mean scores compared with other domains. In Time 1 the average score for close relationship items was 4.5 (out of a possible maximum of 5) whereas the average item score across the 7 domains was 3.24. For Time 2 we therefore sought to devise different items that might not attract this same strong social desirability bias. This resulted in a more diverse set of items that did not correlate so well with each other.

## Conclusion

This paper describes the new approach of IWB, which concerns what people think and feel they are able to be and do. The first part of the paper is conceptual, describing the rationale for IWB, and the process of developing the concept. The second half of the paper is empirical, describing field research in India and the psychometric analysis which resulted in validating the hypothesised seven domain model.

Where wellbeing models are typically developed through research in highly industrialised countries, we were most influenced by research undertaken in and on countries of the global south. A key concern was that our approach to wellbeing should recognise differences by culture and political economy. This shaped the research in two very important ways. Most immediately it informed our methodology. The research was conducted in two very different country locations—one in South Asia and one in sub-Saharan Africa. While recognising that our overall framework would need to be common to both, we were committed to ensuring that the specific questions that we asked would reflect issues of relevance to wellbeing in the particular context of our research. As described briefly above, therefore, the process of developing our quantitative scale to assess IWB was highly qualitative, with a painstaking ongoing process of trying out different questions, listening to how people responded, adjusting and adapting and trying again.

At a deeper level the commitment to being sensitive to cultural difference reflected our understanding of wellbeing as something that is *lived*. While we readily accepted that psychology had been given too little attention in development studies, the insight from WeD that material, relational and subjective dimensions are interwoven aspects of a complex reality made us uneasy with standard psychological approaches that abstracted people’s thinking from their material and social lives. In our view, if subjective perspectives on wellbeing were to express how people were thinking and feeling, then they could not but reflect cultural patterns, political economy and local values. This meant going beyond the standard subjective well-being approach with its ‘thin’ measures of emotional balance and life satisfaction (e.g. Diener et al. 1999) to devise a scale that would capture some, at least, of the substantive realities of people’s lives. Ultimately this aims to propose a different underlying anthropology, challenging the default model of people as consumers rating their happiness or satisfaction with their lives, and restoring participants instead to a more active, embodied subjectivity.

## Footnotes

1 There was some discussion in the group that ‘resources and needs’ are not in fact objective but subjective categories (see e.g. White and Ellison 2007) but this was not taken further within the WeD project.

2 A somewhat similar process is described by Clark (2003) in the context of South Africa.

3 The list is in fact contains approximately one-third of Thailand specific items and two thirds of items common to the other study countries.

4 The term ‘Adivasi’ came into use in post-colonial times (Sundar 2007) and literally means ‘dwellers from the beginning,’ though the legitimacy of this as a historical fact is disputed (e.g. Bates 1995).

5 The fact that most people no longer go hungry is due to a government programme which provides allocations of rice at highly subsidized prices.

6 They mention, for example, that respondents found a seven point response scale difficult to manage, so they shifted to a three point scale, and that the standard correlations found in the Satisfaction With Life Scale did not work for one of the five items (Biswas-Diener and Diener 2001: 342).

7 Prelis is the pre-processing component of LISREL 9.1 (Joreskog and Sorbom 2012a).

8 Values above 2.30 in absolute value generally are considered to be symptomatic of severe non-normality (Lei and Lomax 2005).

9 A full correlation matrix is available from second author upon request.

10 The following combination of fixed and freed parameters was specified: (1) In the measurement error (i.e., theta epsilon, or TE) matrix, all instances of uncorrelated measurement error were freed but were constrained to be equal to each other; all instances of correlated measurement error were fixed at 0.00. (2) In the factor structure (i.e., lambda Y, or LY) matrix, the loadings for all 32 items on a single factor were freed. (3) Finally, the variance–covariance (i.e., psi, or PS) matrix was set to identity (i.e., a single element, fixed at 1.00).

11 The following combination of fixed and freed parameters was specified: (1) In the measurement error matrix, all instances of uncorrelated measurement error among the items that were designed to measure a given factor were freed but were constrained to be equal to each other; all instances of correlated measurement error were fixed at 0.00. (2) In the factor structure matrix, loadings for all items that were hypothesised to load on a given factor were freed; all other loadings were fixed at 0.00. (3) Finally, in the variance–covariance matrix, all factor intercorrelations were freed; all instances of unexplained variance in factor scores were fixed at 1.00.

12 This is a sign of model misspecification for a particular data set (Brown 2006).

13 This is partly because standard errors associated with the loadings were elevated for the eight-factor, non-intercorrelated model, thus reducing the beta/standard error ratios that serve as bases for significance testing in confirmatory factor analysis (Brown 2006).

14 This total excludes one item in the social connections domain that was administered to participants, but not included in Time 2 analyses. It was not included in the analyses because it measures negative social connections, while all the other items measure positive social connections (see also Table 6).

15 A full correlation matrix is available from the second author upon request.

16 The following combination of fixed and freed parameters was specified: (1) In the measurement error (i.e., theta epsilon, or TE) matrix, all instances of uncorrelated measurement error were freed but were constrained to be equal to each other; all instances of correlated measurement error were fixed at 0.00. (2) In the factor structure (i.e., lambda Y, or LY) matrix, the loadings for all 35 items on a single factor were freed. (3) Finally, the variance–covariance (i.e., psi, or PS) matrix was set to identity (i.e., a single element, fixed at 1.00).

17 The following combination of fixed and freed parameters was specified: (1) In the measurement error matrix, all instances of uncorrelated measurement error among the items that were designed to measure a given factor were freed but were constrained to be equal to each other; all instances of correlated measurement error were fixed at 0.00. (2) In the factor structure matrix, loadings for all items that were hypothesised to load on a given factor were freed; all other loadings were fixed at 0.00. (3) Finally, in the variance–covariance matrix, all factor intercorrelations were freed; all instances of unexplained variance in factor scores were fixed at 1.00.

18 Based on standardised factor scores produced by LISREL 9.1.

19 This is due to the ability of confirmatory factor analysis to take measurement error into account when calculating interfactor correlations, thus correcting for attenuation of correlations; for a discussion of the calculation of factor score correlations via confirmatory factor analysis (see Brown and Moore 2012).

20 These are available from the second author upon request.

21 See Kline (2005), concerning desired levels for goodness-of-fit indices and related statistics.

22 See Nunnally and Bernstein (1994), concerning the effects of number of items on scale reliability.

23 This is as distinct from the *absolute* fit of any particular model (Brown 2006).

24 Qualitative data that was collected alongside did not, alas, support this picture of highly harmonious family relationships.
